# Optimizing lung SBRT delivery: A hybrid approach combining dynamic conformal arc (DCA) and volumetric modulated arc therapy (VMAT) techniques

**DOI:** 10.1002/acm2.70217

**Published:** 2025-08-21

**Authors:** Karen Chin Snyder, Amanda DiCarlo, Jessie Huang, Bo Zhao, Yimei Huang, Kundan Thind

**Affiliations:** ^1^ Department of Radiation Oncology Henry Ford Health System Detroit Michigan USA

**Keywords:** delivery, limited optimization, lung SBRT, motion management

## Abstract

**Purpose:**

This study introduces and evaluates a hybrid dynamic conformal arc‐volumetric modulated arc therapy (hDCA‐VMAT) technique for lung stereotactic body radiotherapy (SBRT). The goal is to combine the planning efficiency of VMAT with the delivery robustness of dynamic conformal arc (DCA) techniques, particularly for low‐density lung targets where motion and dose calculation uncertainties pose challenges.

**Methods:**

Twenty‐four previously treated lung SBRT cases were retrospectively replanned using hDCA‐VMAT, conventional VMAT, and aperture controlled VMAT (VMAT_AC). hDCA‐VMAT plans were initiated with a manually created DCA plan, followed by limited inverse optimization with constrained aperture modulation. Plans were created in Eclipse v16.1 and calculated using the AcurosXB algorithm. Dosimetric plan quality, beam complexity, and delivery efficiency were assessed. Complexity was quantified using aperture‐based metrics (e.g., average leaf pair opening, beam area, modulation index). Pretreatment delivery accuracy was evaluated via EPID‐based gamma analysis at 3%/1, 2%/1, and 1%/1 mm Gamma criteria.

**Results:**

All techniques produced clinically acceptable plans. Target coverage and conformity indices were comparable, but hDCA‐VMAT plans demonstrated reduced mid‐dose spread and significantly lower modulation. hDCA‐VMAT achieved the lowest modulation factor (2.1 ± 0.52) and shortest beam on time (1.74 ± 0.46 min), a 27%–30% reduction compared to VMAT and VMAT_AC. Beam complexity metrics confirmed larger, more circular apertures for hDCA‐VMAT. Gamma pass rates were significantly higher for hDCA‐VMAT across all criteria, particularly under stringent 1%/1 mm criteria.

**Conclusion:**

The hDCA‐VMAT technique offers a practical, streamlined approach for lung SBRT planning that reduces modulation while maintaining high plan quality. By initiating with a DCA plan and applying limited optimization only when necessary, hDCA‐VMAT minimizes planning complexity and improves delivery efficiency. These benefits are especially relevant for treating low‐density lung tumors, where robustness to motion and delivery accuracy are critical.

## 1 INTRODUCTION

Lung cancer remains the second most common malignancy among both men and women in the United States, with an estimated 234,580 new cases diagnosed annually.^1^ Stereotactic body radiation therapy (SBRT) has become a cornerstone in the management of early‐stage non‐small cell lung cancer (NSCLC), offering high local tumor control rates with minimal treatment‐related toxicity.^2^ SBRT delivers highly conformal, high‐dose radiation to the target lesion while sparing surrounding healthy tissues, making it a valuable alternative to surgical intervention.

However, delivering SBRT for lung tumors poses significant technical challenges due to respiratory‐induced tumor motion and the heterogeneous nature of lung tissue. Four‐dimensional computed tomography (4DCT) is commonly used to map tumor motion throughout the breathing cycle, but it is prone to errors such as reconstruction artifacts,^3^ patient noncompliance or irregular breathing,^4^, ^5^ and baseline drift over time.^6^ These uncertainties can lead to inaccuracies in dose delivery when highly conformal and modulated plans are based on imaging that may not accurately represent the tumor's density, location, or motion amplitude during treatment.

Volumetric modulated arc therapy (VMAT) is employed by 81% of institutions for lung SBRT, as reported by the American Association of Physicists in Medicine Task Group 324 survey, likely due to its planning and delivery efficiency.^7^ Nonetheless, the increased modulation inherent in VMAT may not be optimal under conditions of significant tumor motion and imaging uncertainties. Simpler techniques such as three‐dimensional conformal radiation therapy (3DCRT) and dynamic conformal arc (DCA) therapy offer more robust delivery by reducing modulation complexity, potentially enhancing treatment accuracy in the presence of motion.^8^ DCA, in particular, aligns multileaf collimator (MLC) apertures closely with the tumor shape throughout the arc, making it less susceptible to the interplay effect between MLC motion and tumor motion. However, DCA planning is a forward process that can be more time‐consuming and skill‐intensive compared to inverse planning methods like VMAT.

Several hybrid techniques have been proposed to balance the planning advantages of modulation with improved dose delivery robustness by combining DCA and VMAT dose delivery,^9^ integration of partial DCA with static beams,^10^ scripting‐based segment optimization,^11^ and the use of DCA apertures to start VMAT optimization.^12^ While effective, these methods often require additional planning time, post‐processing steps, or reliance on institution‐specific scripts.

Minimizing modulation in SBRT delivery is advantageous as it reduces delivery complexity and enhances robustness against uncertainties in MLC modeling and delivery accuracies.^13^ DCA techniques, with their open MLC configurations, produce treatment plans less affected by lung density heterogeneities and errors in 4DCT reconstruction.^14^, ^15^ Further complicating the matter is the adoption of more accurate dose calculation algorithms, such as grid‐based Boltzmann solvers (AcurosXB) or Monte Carlo methods.^16^, ^17^, ^18^ The increased accuracy that these algorithms offer over simplified algorithms used in the optimization process results in larger discrepancies between the optimized plan and the final dose calculation. These discrepancies can make it challenging to achieve full tumor coverage, particularly at the periphery, due to lower calculated doses at the lung‐tissue interface.^19^ Compensating for this effect often necessitates increased modulation to deliver additional fluence to the tumor edges, complicating the planning process.

This study introduces and evaluates a hybrid dynamic conformal arc–volumetric modulated arc therapy (hDCA‐VMAT) technique for lung SBRT, with the objective of integrating VMAT's planning efficiency with DCA's inherent delivery robustness. Adapted from a cranial radiosurgery approach,^20^ this method utilizes a foundational DCA plan which then undergoes limited inverse VMAT optimization. Unlike the aforementioned methods that combine different techniques or utilizes external software, the inverse VMAT optimization only needs to be used if the foundational DCA plan needs additional modification. We compare the dosimetric characteristics, modulation complexity, and estimated robustness of hDCA‐VMAT plans against both traditional VMAT and an aperture‐controlled VMAT (VMAT_AC) technique. We hypothesize that both hDCA‐VMAT and VMAT_AC techniques can produce treatment plans with enhanced plan robustness and reduced modulation compared to traditional VMAT plans, while maintaining comparable treatment plan quality.

## 2 METHODS AND MATERIALS

### 2.1 Patient selection and characteristics

Twenty‐four lung cancer patients previously treated at our institution with SBRT were included in this IRB approved retrospective study [approval 12934]. All patients underwent a free breathing 4D CT simulation (Philips Big Bore, Amsterdam). The 4DCT was reconstructed using 10 phases and evaluated by a qualified medical physicist to ensure appropriate image quality and to identify artifacts that may interfere with target delineation. A motion encompassing ITV based approach was used where an average intensity projection (AIP) image was reconstructed for treatment planning and dose calculation. The ITV was contoured based on the maximum intensity project (MIP) and verified on the 10 phases. The planning target volume (PTV) was generated by the physician by adding a 5 mm uniform margin around the ITV. The mean PTV volume was 25.69 cc (range 5.68–79.82 cc) and mean PTV HU was −631 HU (range −842 to −272 HU).

### 2.2 Plan characteristics

Treatment planning was performed in the Eclipse treatment planning system v 16.1 (Varian, Palo Alto, CA, USA). Plans were optimized for an EDGE linear accelerator equipped with a high‐definition MLC (HD‐MLC) utilizing the 6× flattening filter free (FFF) beam with 1400 MU/min dose rate. Plans were calculated utilizing AcurosXB v15.6.05 with the default calculation grid size of 1.25 mm for FFF stereotactic beams and optimized using the photon optimizer (PO) v 16.1.12.

Plans consisted of 2–4 partial, non‐coplanar arcs. The number of arcs used was determined by the location of the lesion‐where two partial arcs were used for centrally located, island type lesions, whereas a posterior lesion required additional small partial arcs to help with conformality. The prescription dose varied from 50 to 60 Gy in five fractions (10–12 Gy per fraction). Plans were normalized per RTOG 0813 guidelines so that no less than 95% of the target volume received 100% of the prescription dose.^21^

### 2.3 Hybrid DCA‐VMAT plans

The hybrid dynamic conformal arc‐VMAT (hDCA‐VMAT) plans consisted of a manually planned dynamic conformal arc (DCA) plan which was the starting point for a limited inverse optimization. Initial DCA plans were created by shaping the MLCs of each beam to the PTV from the beams‐eye‐view (BEV). The initial margin from the MLCs to the target volume was 0 mm in the axial direction, and 3 mm in the longitudinal axis. After dose calculation with the AcurosXB algorithm, the arcs were re‐weighted to create a spherical dose distribution. Plans were normalized to 100% at target dose maximum and a prescription isodose line was chosen around 70%–80%. If necessary, an optimization planning target volume (optPTV) was created to fine‐tune the conformality of the dose distribution; where portions of the optPTV were added or subtracted to increase and decrease the PTV coverage, respectively. Once the conformality was improved, without violation of RTOG 0813 gradient criteria, the plan was normalized so that ≥95% of the target volume was covered by the prescription volume.

To enforce limited inverse optimization, the aperture control feature of the photon optimizer (PO) was utilized, and the optimization focused exclusively on the final resolution levels. The aperture control setting was configured to “very high” to minimize modulation and preserve the larger MLC apertures derived from the initial DCA plan. The hybrid optimization proceeded iteratively, starting at multi‐resolution 4 (MR4), the highest resolution level of the PO optimizer. At this stage, MLC movements are tightly constrained, allowing adjustments only within the operational limits of the treatment machine, such as maximum gantry, leaf, jaw speed. If the optimization failed to meet the prescribed dose constraints at MR4, the process was adjusted by stepping back to multi‐resolution 3 (MR3). MR3 offers fewer restrictions on MLC motion compared to MR4, granting additional flexibility for modulation while still maintaining the foundational MLC positions from the DCA plan. This iterative approach allowed for controlled modulation adjustments, ensuring that dose constraints were met without introducing excessive complexity to the MLC configurations. Detailed procedural instructions for this optimization method are provided in the Supplementary materials.

### 2.4 VMAT plans

hDCA‐VMAT plans were compared to conventional VMAT and aperture controlled VMAT (VMAT_AC) plans to evaluate the change in modulation. The same plan geometry (gantry, couch, collimator angles) was utilized for VMAT optimization. For consistency, an in‐house knowledge‐based RapidPlan model based on the work performed by Snyder et al. was used to optimize the VMAT plans.^22^ The VMAT plans utilized a 4‐ring technique, to control the hotspot, conformality, and gradient indices. A full inverse optimized VMAT plan was first created with no limitations on monitor units or aperture shape. Utilizing the same objectives and constraints from the VMAT plan, another VMAT plan (VMAT_AC) was created setting the aperture control to “Very High”. All VMAT plans (VMAT and VMAT_AC) plans were calculated using the AcurosXB algorithm.

### 2.5 Plan quality, modulation and beam complexity evaluation

The plan quality, beam modulation, and beam on time were evaluated for all plans. Dose fall off criteria was evaluated following RTOG 0813 criteria^21^, ^23^, ^24^ including conformity index (CI), Paddick gradient index (GI) and dose gradient criteria including the maximum dose 2 cm away from target (D_nonPTV + 2 cm) and outside the target volume (D_nonPTV). The Paddick Conformity Index^25^ was also calculated to ensure the value was above 0.85, and compared amongst the plans. OAR doses were evaluated using AAPM TG 101 constraints for normal dose tolerances for Lung SBRT treatment in 5 fractions.

Beam complexity was evaluated using an aperture based approach with metrics conceived and characterized by Du et al., utilizing open source ESAPI Script.^26^, ^27^ For each plan, the average leaf pair opening (ALPO), beam area (BA), beam modulation (BM), and beam irregularity (BI) were calculated and compared between the hDCA‐VMAT, VMAT, and VMAT_AC plans. The definition and derivation of these metrics are detailed in the work by Du et al., and a brief summary is provided here for reference. The average leaf pair opening (ALPO) represents the mean distance between opposing MLC leaf pairs in mm, average over all control points in the plan. The beam area (BA) is the MLC aperture area at each control point, weighted by MU, then average across all control points; the larger BA values correspond to more open aperture. The beam modulation (BM) is defined as one minus the ratio of the average MU weighted aperture area per control to the union area of all apertures within the beam. Values range from 0 to 1, where a static MLC plan, yields a BM of 0, while highly modulated beams with many small, spatially seperated apertures results in BM value approaching 1. The beam irregularity (BI) describes the circularity of the beam aperture. For a circular aperture BI equals 1. Larger values indicate increasingly narrow or irregular shaped apertures.

More commonly used metrics including the modulation factor (MF), monitor units (MU), and beam on time were also calculated for all plans. The modulation factor (MF) is the ratio of the total plan monitor units divided by the dose per fraction in cGy. Beam on time was calculated by the treatment planning system based on the gantry speed and control points and did not include the amount of time to move from field to field. Plan quality criteria, modulation factor, and beam on time were statically evaluated for significant differences using a non‐parametric Kruskal–Wallis test with Bonferroni correction, followed by post‐hoc Dunn's test for pairwise multiple comparisons, where a p‐value less than 0.05 was considered statistically significant. (MATLAB R2024b, The MathWorks Inc., Natick, MA, USA).

### 2.6 Pretreatment delivery

Pretreatment quality assurance (QA) was performed on all plans to validate plan delivery. QA was performed using Sun Nuclear PerFraction v2.11.0 (Sun Nuclear Corporation, Melbourne, FL), electronic portal imaging device (EPID) based pre‐treatment QA (Fraction 0). All plans were delivered on the EDGE linear accelerator equipped with an a‐Si 1200 EPID with a 40 × 40 cm^2^ with an 1190 × 1190 pixel array. 2D absolute dose comparison with the predicted dose was completed on a field‐by‐field basis for each plan. Gamma analysis using global normalization with a dose threshold of 10% and dose difference/distance‐to‐agreement criteria of 3%/1, 2%/1, and 1%/1 mm were used to evaluate the accuracy of delivery. The three plans: hDCA‐VMAT, VMAT_AC, and VMAT were delivered for each patient, resulting in 52 per‐field gamma analyses for each planning technique. Mean gamma passing rates over all individual fields and all patients were compared between planning techniques using a Kruskal–Wallis test with Bonferonni correction with post‐hoc Dunn's test.

## 3 RESULTS

### 3.1 Plan quality

**FIGURE 1:**
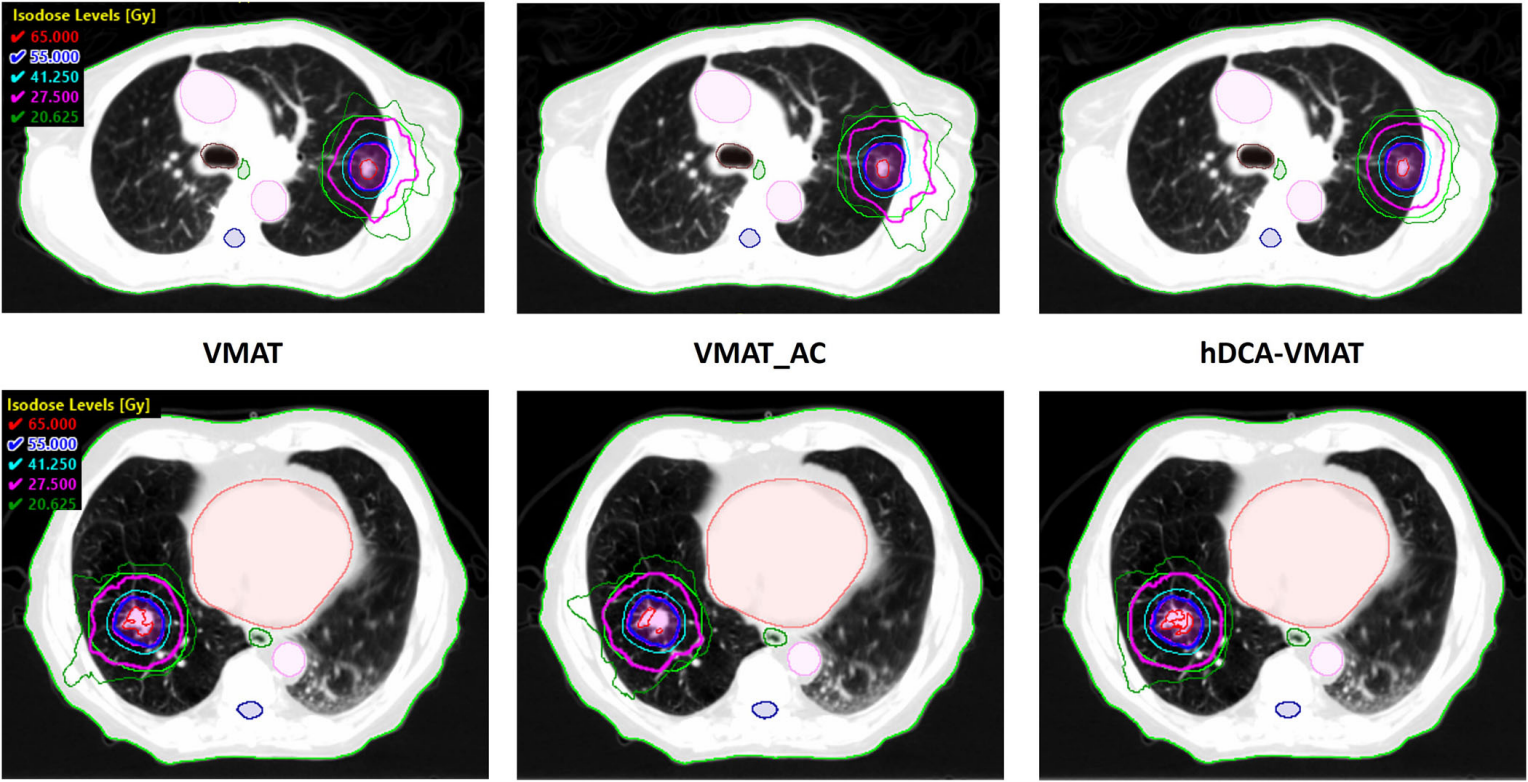
Axial view of dose distributions for VMAT, VMAT_AC, and hDCA‐VMAT plans showing an example of a lateral lesion next to the rib (top) and an island type lesion (bottom) surrounded by lowdensity lung tissue. Target (magenta) and 2 cm ring (green) shown relative to isodose lines: 100% prescription (blue), 50% prescription (magenta). Along with OARs such as heart (orange), esophagus (dark green), great vessel (pink) and spinal cord (dark blue). Note changes in conformality and smoothness of the high and low isodose lines.

Table 1 summarizes the results of target coverage, conformality and OAR doses for the three planning techniques. For all plans, no significant difference in target coverage of PTV_D99% or conformality, evaluated using RTOG CI, Paddick CI, GI, and D_nonPTV, was observed between hDCA‐VMAT, VMAT_AC, and VMAT plans, *p *> 0.05. Differences in the mid‐dose spread quantified by D_nonPTV+2 cm were observed. The hDCA‐VMAT plans resulted in less mid dose spread with an average nonPTV+2 cm value 2 Gy less than the VMAT and VMAT_AC plans. Figure 1 demonstrates axial views of dose distributions for VMAT, VMAT_AC, and hDCA‐VMAT plans for two common scenarios, a lateral lesion next to the rib and an island type lesion surrounded by low density lung tissue. It can be observed that when heterogeneities such as the rib or large amounts of soft tissue are adjacent to the lesion, the low dose isodose lines increase in the fully inverse optimized VMAT plan, while the hDCA‐VMAT plans maintain a more spherical dose distribution. Similar behavior can also be observed for island‐type lesions, though to a lesser extent.

**TABLE 1:** Plan quality metrics and OAR doses for all patients for three different planning techniques. Mean ± standard deviation (minimum–maximum) values are reported.

Parameter	VMAT	VMAT_AC	hDCA‐VMAT	*p*‐value
PTV				
D_99%_ (Gy)	53.6 ± 3.6 (47.4–60.4)	53.7 ± 3.6 (48.1c60.2)	53.7 ± 3.5 (48.3–59.2)	n.s.
D_max_ (Gy)	63.8 ± 4.7 (58.4–76.5)	67.1 ± 4.7 (58.3–75.2)	67.5 ± 5.0 (55.7–75)	n.s.
Conformality				
GI	4.61 ± 1.39 (3.14–8.56)	4.63 ± 1.38 (3.21–8.61)	4.75 ± 1.36 (3.24–8.76)	n.s.
CI	1.02 ± 0.07 (0.95–1.19)	1.03 ± 0.08 (0.96–1.21)	1.04 ± 0.06 (0.97–1.26)	n.s.
Paddick CI	0.89 ± 0.06 (0.76–0.95)	0.89 ± 0.06 (0.75–0.95)	0.89 ± 0.05 (0.72–0.94)	n.s.
nonPTV	0.01 ± 0.02 (0–0.08)	0.02 ± 0.03 (0–0.08)	0.02 ± 0.03 (0–0.15)	n.s.
nonPTV+2 cm (Gy)	28.5 ± 4.0 (20.2–41.3)	28.4 ± 3.9 (19.4–38.4)	26.7 ± 3.9 (19.0–39.0)	^*^, ^**^
Spinal cord				
D_0.35cc_ (Gy)	7.7 ± 4.1 (2.2–16.2)	7.6 ± 4.4 (2.1–17.2)	7.5 ± 4.0 (1.8–15.2)	n.s.
D_0.035cc_ (Gy)	8.6 ± 4.6 (2.3–18.9)	8.4 ± 4.9 (2.3–19.1)	8.1 ± 4.4 (1.9–17.6)	n.s.
Esophagus				
D_5cc_ (Gy)	3.8 ± 3.4 (0.6–15.8)	3.8 ± 3.8 (0.5–18.5)	4.0 ± 3.8 (0.5–18.5)	n.s.
D_0.035cc_ (Gy)	10.0 ± 5.9 (2.3–31.5)	9.8 ± 6.0 (27–31.2)	9.6 ± 6.0 (2.3–31.6)	n.s.
Heart				
D_15cc_ (Gy)	6.0 ± 6.0 (0.1–17.8)	6.0 ± 6.1 (0.1–18.5)	5.7 ± 5.6 (0.1–16.7)	n.s.
D_0.035cc_ (Gy)	10.8 ± 10.9 (0.2–39.25)	10.7 ± 10.8 (0.2–38.5)	9.9 ± 9.8 (0.2–33.8)	n.s.
Rib				
D_1cc_ (Gy)	32.1 ± 14.1 (9–57.7)	32.5 ± 14.2 (8.8–57.7)	31.5 ± 14.2 (9.2–55.5)	n.s.
D_0.035cc_ (Gy)	37.9 ± 14.8 (16.5–59.7)	38.1 ± 15.0 (17.9–59.7)	36.5 ± 14.9 (15.4–58.9)	n.s.
Skin				
D_10cc_ (Gy)	10.6 ± 3.4 (5.9–20.2)	10.6 ± 3.4 (5.8–20.6)	10.4 ± 3.5 (5.7–21.7)	n.s.
D_0.035cc_ (Gy)	18.8 ± 5.1 (10.7–31.1)	18.4 ± 5.0 (10.1–30.5)	16.9 ± 5.9 (8.8–35.8)	^*^
Modulation				
MU	3353 ± 593 (2272–4867)	3120 ± 650 (2126–4720)	2337 ± 683 (1536–4106)	^*^, ^**^
MF	3.0 ± 0.5 (2.2–6.2)	2.8 ± 0.5 (2.1–4.3)	2.1 ± 0.6 (1.5–3.7)	^*^, ^**^, ^***^
Beam on time (min)	2.4 ± 0.43 (1.64–3.48)	2.2 ± 0.47 (1.52–3.37)	1.74 ± 0.46 (1.1–2.97)	^*^, ^**^, ^***^

* *p*‐value < 0.0005 for hDCA‐VMAT compared to VMAT.

** *p*‐value < 0.0005 for hDCA‐VMAT compared to VMAT_AC.

*** *p*‐value < 0.05 for VMAT compared to VMAT_AC.

Only two cases were outside RTOG 0813 criteria, where the GI values would be considered a major protocol deviation. It was noted that the two cases had low density targets, with average PTV HUs values less than −794 HU. One of the two cases was an island type lesion, with an average PTV HU of −839 HU, also resulted in a major protocol deviation of the D_nonPTV+2 cm criteria for all plans.

### 3.2 Beam modulation

**FIGURE 2:**
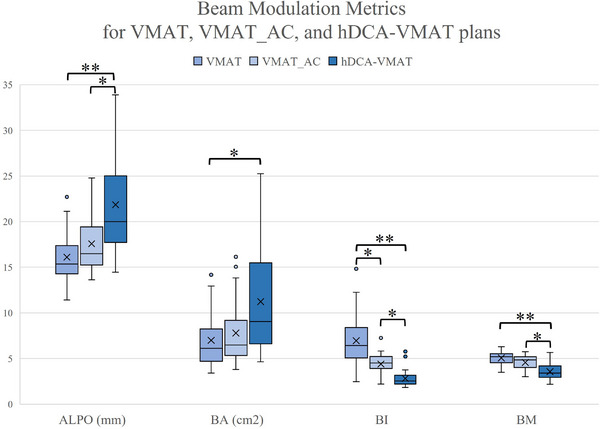
Box and whisker plots depicting beam modulation metrics for VMAT, VMAT_AC, and hDCA‐VMAT plans. Average leaf pair opening (ALPO) is the average distance between open leaf pairs and Beam Area (BA) is the total area of all MLC openings in a field averaged over all the fields. Less modulation is demonstrated by larger ALPO and BA values. Beam Irregularity (BI) describes the circularity of the beam aperture. Beam Modulation (BM) is 1 minus the ratio of beam area normalized to the sum of the total area per beam. Larger, circular fields are demonstrated by smaller BI and BM values. Asterisks indicate statistical significance between the planning techniques (*p < 0.05 and **p < 0.0005).

Figure 2 summarizes the beam aperture and modulation metrics for the 3 different techniques. hDCA‐VMAT resulted in the largest beam aperture of the three techniques, with greater ALPO, AA, and BA values compared to VMAT and VMAT_AC. hDCA‐VMAT also resulted in lower BI and BM metrics than both the VMAT and VMAT_AC plans, demonstrating hDCA‐VMAT plans' similarity to more circular, 3D conformal fields due to their derivation from DCA plans. Aperture controlled VMAT beam modulation metrics fell between hDCA‐VMAT and VMAT techniques, however were more similar to the VMAT plans than the hDCA‐VMAT.

Table 1 summarizes the MU, MF and beam on time results. The average MUs of hDCA‐VMAT was less than the average MUs of VMAT and VMAT_AC plans. The average modulation factor for hDCA‐VMAT was less than both the VMAT and VMAT_AC plans. The MF for VMAT_AC was also found to be statistically less than VMAT plans. hDCA‐VMAT resulted in faster beam on times, with a 27–30% reduction in time compared to VMAT and VMAT_AC plans. Similarly, the VMAT_AC plans required less beam on time than the non‐aperture controlled VMAT plans.

**FIGURE 3:**
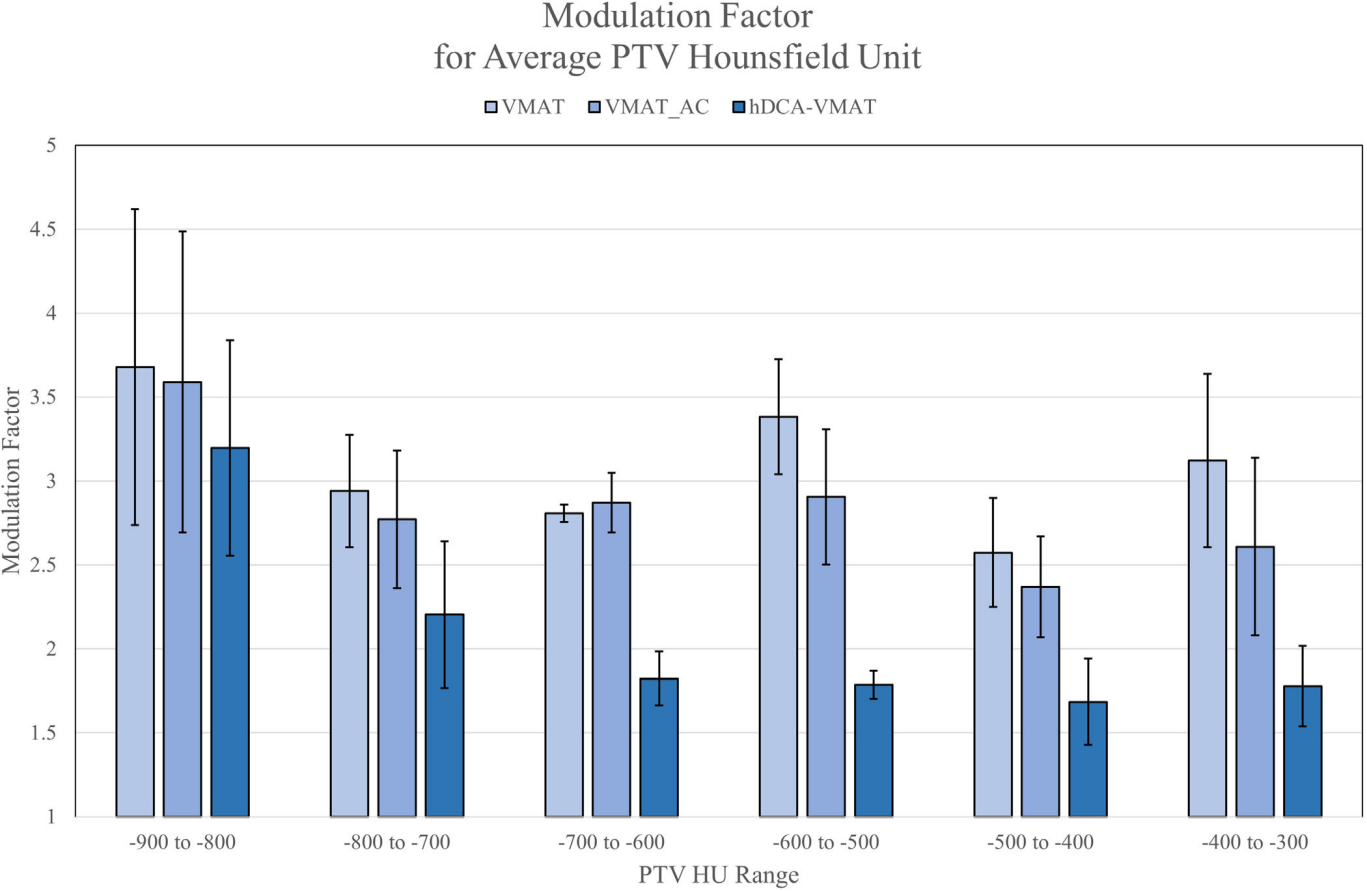
Modulation Factor (MF) for hDCA‐VMAT, VMAT_AC, and VMAT plans relative to average HU value of the PTV over the patient cohort. Average values are shown with standard deviation in error bars.

When the PTVs were stratified based on the average HU value into six evenly distributed groups (−900 to −300 HU), the MF for hDCA‐VMAT, VMAT_AC, and VMAT plans tended to increase as the target density decreased, approaching the value of air (−1000 HU), Figure 3. The hDCA‐VMAT MF in all HU groupings were less than both the VMAT and VMAT_AC. The difference was greater for the average PTV HU value of −700 to −300, but over the patient cohort, the VMAT MF was 1.5 times greater than the hDCA‐VMAT plans.

### 3.3 Delivery

**FIGURE 4:**
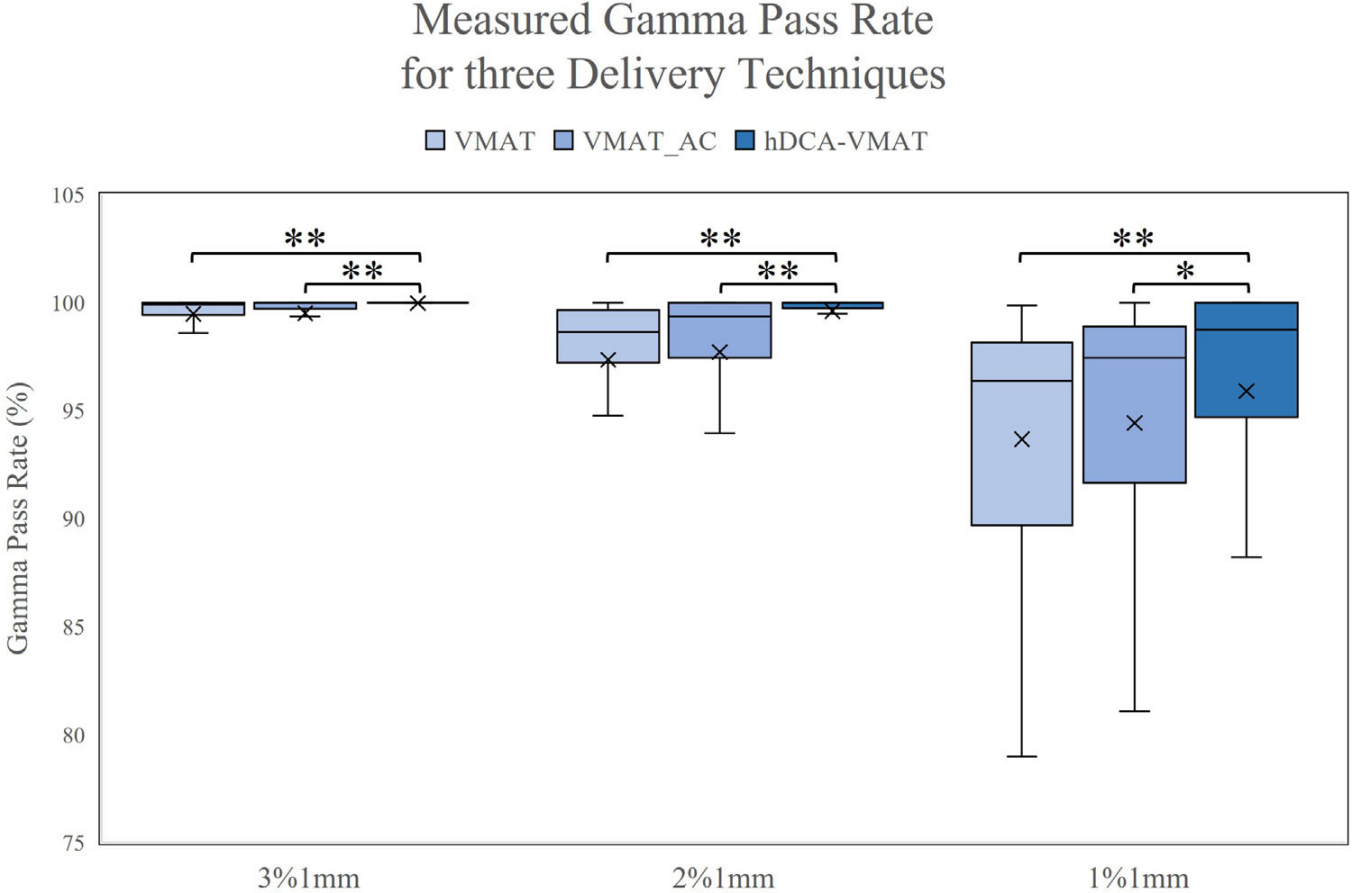
Box and whisker plots depicting differences in sensitivity of Gamma Analysis for VMAT, VMAT_AC, and hDCA‐VMAT plans for gamma criteria of 3%1 mm, 2%1 mm, and 1%1 mm. Asterisks indicate statistical significance between the delivery techniques (*p < 0.05 and **p < 0.0005).

Pre‐treatment QA results are summarized in Figure 4. Utilizing 3%1 mm gamma criteria, all fields across all techniques achieved a gamma passing rate of >90%. When the dose difference criteria were further restricted to 2%, all fields planned with hDCA‐VMAT passed gamma analysis (min. 97% pass rate), while three fields planned with VMAT and one field planned with VMAT‐AC failed gamma analysis (gamma pass rate < 90%). If a very stringent gamma criteria of 1%1 mm was used, 11.5%, 21.2%, and 15.3% of fields planned with hDCA‐VMAT, VMAT, and VMAT_AC, failed gamma analysis at a pass rate of 90%. When evaluating the mean gamma pass rates for all fields, a significant difference (*p* < 0.0005) was found between deliverability of hDCA‐VMAT plans versus VMAT plans for gamma criteria of 3%1, 2%1, and 1%1 mm. No difference was observed in gamma criteria pass rate comparing VMAT to VMAT_AC. Of all individual points failing gamma analysis, the vast majority measured doses lower than the treatment plan.

## 4 DISCUSSION

Delivering dose to low‐density lung tissue presents significant dosimetric and planning challenges including achieving target coverage while maintaining a steep dose falloff, minimizing treatment delivery time, reducing modulation to achieve a plan robust to patient respiratory motion as well as ensuring deliverability. To this end, our study investigates the use of a DCA forward planned technique as a foundation for constrained optimization compared to conventional inverse optimized techniques.

The advantage of VMAT is the ability to use inverse optimization to achieve the planning goals, whereas forward planned DCA requires more planner skill, manual plan adjustment, and planning time.^8^ Our proposed hybrid planning technique in which DCA plans undergo a limited optimization, is a potential compromise between the two techniques. Advantages of the hDCA‐VMAT technique are plans that are faster to deliver (72% decrease in beam on time compared to VMAT), less modulated (larger apertures and lower beam modulation metrics compared to VMAT), and delivery with better dosimetric agreement with the planning system (higher gamma passing rates for EPID‐based patient specific QA). Our study also included VMAT_AC plans, which exhibit modulation characteristics between the two techniques. The allure of faster, less modulated treatments resulting in better delivery accuracy for lung SBRT treatments has also encouraged other commercial treatment planning systems, such as Raystation, to investigate methods of utilizing more DCA based lung SBRT planning such a their segment weighted optimized dynamic conformal arc (SWO‐DCA).^13^ In comparing our hybrid planning technique with the study by Pokhrel et al., who also combined DCA MLC apertures and VMAT optimization, our plans resulted in slightly less plan modulation (MF of 2.1 vs. 2.3), indicating that our plans may be more similar to DCA.^12^ However, as demonstrated in this study, these differences in plan complexity may also be due to differences in dose calculation and characteristics of the patient cohort (i.e., location, size, and density of tumors).

In the hDCA‐VMAT technique, inverse optimization begins from the DCA plan at MR4, where MLC and gantry movements are tightly constrained within the mechanical operational limits of the machine, resulting in minimal additional modulation. For PTVs with low average HU values, achieving adequate dose coverage may require additional modulation; in such cases, the planner may step back to MR3, which allows for increased MLC motion. Notably, as the optimization process regresses to lower resolution levels, the plan's modulation characteristics more closely align with those of the VMAT_AC plan, highlighting the balance between minimizing modulation for robustness and introducing necessary complexity to meet dose objectives in challenging scenarios.

Forward planning becomes challenging when nearby OARs or irregularly shaped targets are present. In this study, we evaluated VMAT_AC for its ability to reduce MLC modulation by using larger apertures, while simultaneously employing the inverse planning technique with aperture constraints to minimize reliance on the planner's expertise in designing the initial DCA plan. The MF and beam on time of VMAT_AC plans were more similar to standard VMAT plans than to hDCA‐VMAT plans. Notably, hDCA‐VMAT plans were approximately 27% faster than both VMAT and VMAT_AC plans, attributed to the original DCA plans utilizing and maintaining the maximum gantry speed during MR4 inverse optimization. The decreased modulation in hDCA‐VMAT plans may also improve the robustness of treatment delivery such as MLC positioning and MLC interplay effects with respiratory motion.^28^, ^29^

The RTOG intermediate dose compliance criteria were established using convolution/superposition dose calculation algorithms and after evaluation using Monte Carlo, it has been suggested that the compliance criteria may need to be loosened for more accurate algorithms that better model electron disequilibrium.^18^ With more accurate dose algorithms, it becomes challenging to maintain a steep dose fall off in the low density lung while also maintaining a reasonable hotspot (120%) without significant modulation.

In our study, we use the average PTV HU to stratify low‐density lesions. However, this value can be influenced by several factors. The presence of nearby rib or other tissue in the setup margin between the ITV and PTV, as well as amount of tumor motion, which may increase the inclusion of normal lung tissue in the ITV and decrease the average HU. While there is no definitive threshold for defining “low density”, normal lung tissue typically measures around ‐789 HU, whereas emphysematous lung approaches −900.^30^ When the PTV includes both the lesion and adjacent lung tissue, the mean HU will generally increase due to the higher density of the lesion. In our cohort, PTV mean values of −700 to −900 were not uncommon. The observed reduction in MF within this HU range suggests a potential advantage of the hDCA‐VMAT over conventional VMAT planning techniques to minimize MUs and modulation for patients with low density targets.

**FIGURE 5:**
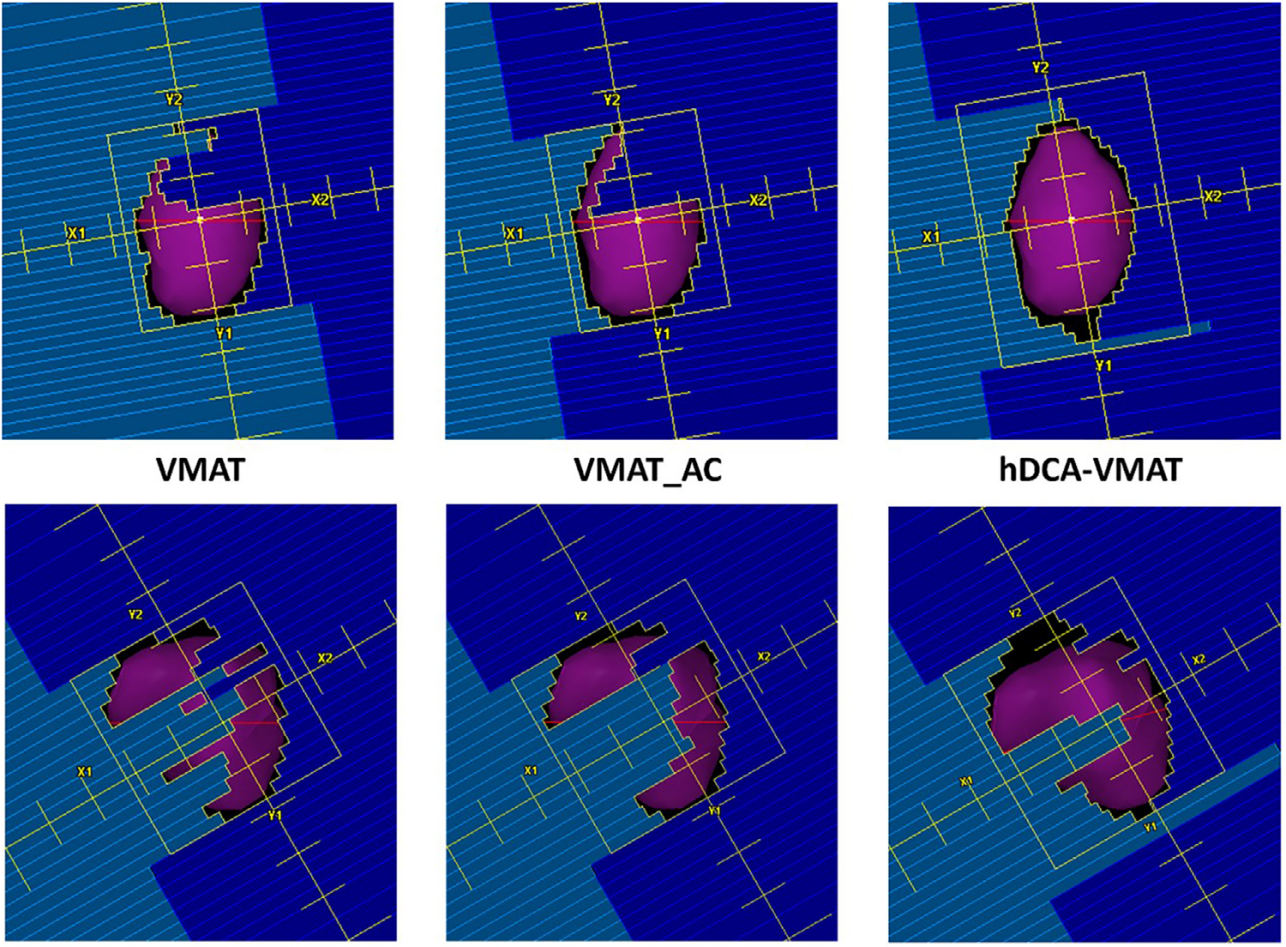
Beams eye view of beam aperture from the same control point of a VMAT, VMAT_AC, and hDCA‐VMAT plan demonstrating the small MLC shapes formed in the VMAT plan, to larger open MLC shapes in the aperture controlled VMAT plan and even larger open MLC shapes in the hDCA‐VMAT plan.

Our study evaluated the dosimetric accuracy of the treatment plans by using our institution's procedure for plan specific QA. A potential reason patient‐specific QA results were better for hDCA‐VMAT than the VMAT plans, is the size and complexity of the modulation between control points and thus the lower modulation of the hybrid plans. Utilizing DCA plans as the starting point for inverse optimization results in more spherical MLC shapes with less interleaf digitation, decreasing the uncertainty of small field output factors. Figure 5 shows the BEV from the same gantry angle for a VMAT, VMAT_AC, and hDCA‐VMAT plan demonstrating the differences in the small MLC apertures between the techniques. The VMAT_AC plan utilizing the very‐high aperture control results in MLC shapes similar to that of the VMAT plan, but with less small MLC opening resulting from MLC digitation. This is also justified quantitatively with larger ALDO and BA values for the VMAT_AC plans compared to the VMAT plans. However, the hDCA‐VMAT plans result in the largest MLC apertures compared to both VMAT and VMAT_AC plans.

The 3%1 mm gamma criteria used in this study was selected to match that used clinically at our institution for pretreatment QA of SRS/SBRT. However, utilizing tighter dose constraints of 2%/1 and 1%1 mm further demonstrated the delivery robustness between the techniques. Ge et al. compared the dosimetry, delivery accuracy and interplay effect of lung SBRT plans with high and low complexity (MU restriction).^31^ Using a respiratory motion phantom and microdiamond detector, they found no substantial dose deviation due to motion for high or low complexity plans. More investigation would be required to understand if the higher passing rates observed in our hDCA‐VMAT plans translate to more accurate treatments in scenarios that more closely mimic patient treatments.

Our proposed technique distinguishes itself from other hybrid approaches by eliminating the need for additional scripting, third‐party software, or specialized planning licenses. The hybrid DCA‐VMAT (hDCA‐VMAT) method integrates seamlessly into an efficient planning workflow that prioritizes reduced modulation. Because the process begins with a dynamic conformal arc (DCA) plan, cases that meet clinical objectives without the need for inverse optimization can be completed with minimal complexity. In instances where the DCA plan alone does not achieve adequate target coverage or OAR sparing, the hybrid method can be applied iteratively to improve dosimetry. Future directions include developing an automated planning pipeline for lung SBRT that initiates with a DCA plan and selectively applies hDCA‐VMAT refinement as necessary.

## 5 CONCLUSION

In this study we have described a hybrid dynamic conformal arc‐VMAT technique and demonstrated that this technique can achieve equivalent plan quality with significantly lower modulation than VMAT plans, specifically for PTV volumes of lower density. The proposed technique utilizes a workflow that emphasizes decreased modulation by initiating with DCA planning; limited inverse optimization is applied when additional modulation is required to meet dose compliance criteria. This allows for efficiency in planning and delivery while maintaining plan quality. The reduction in modulation correlates with statistically significant better gamma pass rate and faster beam on times, thereby optimizing lung SBRT treatment delivery.

## AUTHOR CONTRIBUTIONS

Karen Chin Snyder: Designed; directed the project; and wrote the manuscript. Amanda DiCarlo: performed measurements and analyzed data. All authors provided critical feedback and helped shape the research and final manuscript.

## CONFLICT OF INTEREST STATEMENT

The authors declare no conflicts of interest.

## Supporting information



Supporting information

## Data Availability

Research data are stored in an institutional repository and will be shared upon request to the corresponding author.
