# Supplementary material for: Optimizing lung SBRT delivery: A hybrid approach combining dynamic conformal arc (DCA) and volumetric modulated arc therapy (VMAT) techniques
Source: J Appl Clin Med Phys. 2025 Aug 21;26(9):e70217. doi: 10.1002/acm2.70217 (PMC12795709; doi:10.1002/acm2.70217)
Supplement: Supplementary file 1 — Supporting information [file ACM2-26-e70217-s001.pdf]

How To: Hybrid Arc

Karen Chin Snyder, 2023

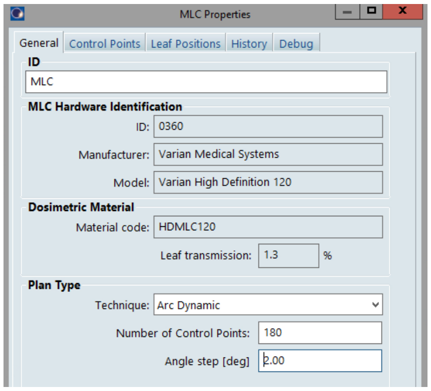

The Hybrid Arc technique can be used in the treatment planning for lung SBRT treatments or for small lesions, where modulation would like to be kept to a minimum, ie Breath Hold, ABC, large amount of motion.

1. Start off with a Dynamic Conformal Arc (DCA) plan that is reasonable (tweak MLCs and reweigh arcs) Renormalize as necessary to achieve plan objectives.
    a. Insert new MLC on an SRS-ARC beam.
    b. Use Technique: Arc Dynamic, Angle step [deg] = 2 or less
    c. Shape MLC. RMC on MLC and "Fit to structure". Set your PTV as the target structure.
    d. Margin: Cranial SRS: 0.1cm circular. Lung SBRT: 0cm R/L, 0.3cmm S/I

    Note: if you change any beam geometry, the MLCs must be refitted manually!

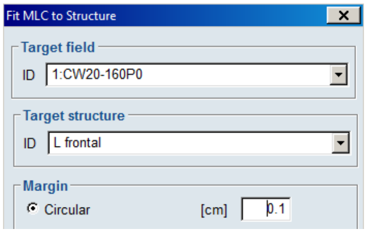

2. After dose calculation, change the calculation option of the PO optimizer. Calculation Models > Edit . Change the Aperture shape controller to "Very High".

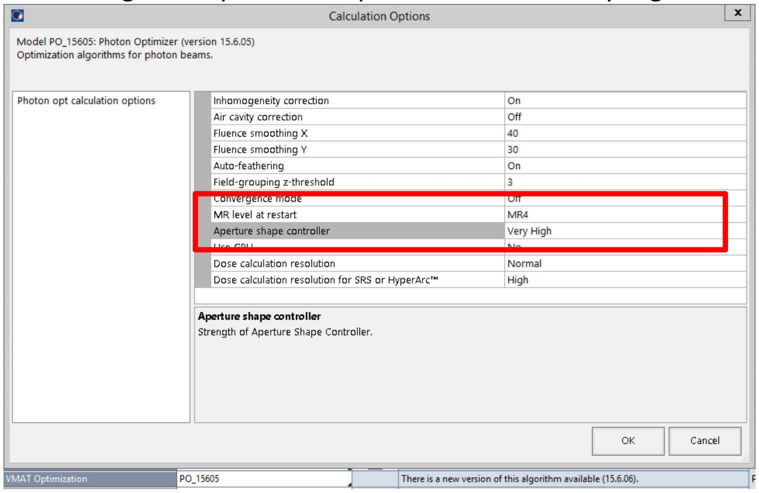

3. Calculate dose with preset values [Shift+F5], or Planning > Dose Calculation > Calculate with Preset Values.
4. The MUs should be saved from the previous calculation. Change the Plan Normalization Value to 100%. Click "OK". MUs should not change.

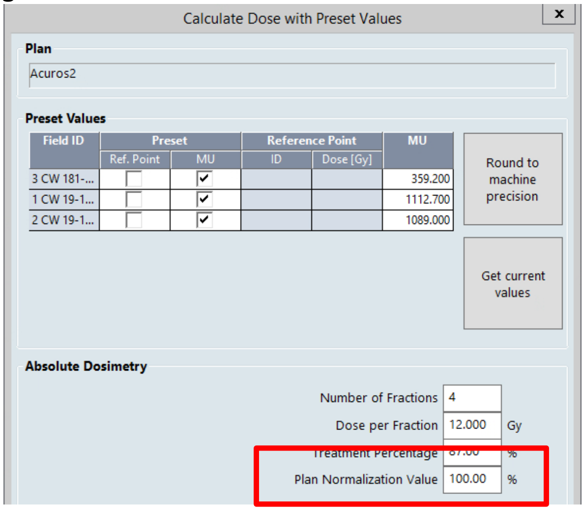

5. Go to optimization workspace. Place priority and objectives as needed, for both upper and lower constraints.
6. Start optimization. Continue from the previous optimization.
7. If MR 4 is not enough to obtain sufficient plan quality, you can step back into MR 3. Stepping back into MR1 or 2 is not recommended.
